# Analysis of gene expression profiles in HeLa cells in response to overexpression or siRNA-mediated depletion of NASP

**DOI:** 10.1186/1477-7827-7-45

**Published:** 2009-05-13

**Authors:** Oleg M Alekseev, Richard T Richardson, Oleg Alekseev, Michael G O'Rand

**Affiliations:** 1 Department of Cell and Developmental Biology, University of North Carolina at Chapel Hill, Chapel Hill, NC, 27599-7090, USA

## Abstract

**Background:**

NASP (Nuclear Autoantigenic Sperm Protein) is a linker histone chaperone required for normal cell division. Changes in NASP expression significantly affect cell growth and development; loss of gene function results in embryonic lethality. However, the mechanism by which NASP exerts its effects in the cell cycle is not understood. To understand the pathways and networks that may involve NASP function, we evaluated gene expression in HeLa cells in which NASP was either overexpressed or depleted by siRNA.

**Methods:**

Total RNA from HeLa cells overexpressing NASP or depleted of NASP by siRNA treatment was converted to cRNA with incorporation of Cy5-CTP (experimental samples), or Cy3-CTP (control samples). The labeled cRNA samples were hybridized to whole human genome microarrays (Agilent Technologies, Wilmington, Delaware, USA). Various gene expression analysis techniques were employed: Significance Analysis of Microarrays (SAM), Expression Analysis Systematic Explorer (EASE), and Ingenuity Pathways Analysis (IPA).

**Results:**

From approximately 36 thousand genes present in a total human genome microarray, we identified a set of 47 up-regulated and 7 down-regulated genes as a result of NASP overexpression. Similarly we identified a set of 56 up-regulated and 71 down-regulated genes as a result of NASP siRNA treatment. Gene ontology, molecular network and canonical pathway analysis of NASP overexpression demonstrated that the most significant changes were in proteins participating in organismal injury, immune response, and cellular growth and cancer pathways (major "hubs": TNF, FOS, EGR1, NFκB, IRF7, STAT1, IL6). Depletion of NASP elicited the changed expression of proteins involved in DNA replication, repair and development, followed by reproductive system disease, and cancer and cell cycle pathways (major "hubs": E2F8, TP53, FGF, FSH, FST, hCG, NFκB, TRAF6).

**Conclusion:**

This study has demonstrated that NASP belongs to a network of genes and gene functions that are critical for cell survival. We have confirmed the previously reported interactions between NASP and HSP90, HSP70, histone H1, histone H3, and TRAF6. Overexpression and depletion of NASP identified overlapping networks that included TNF as a core protein, confirming that both high and low levels of NASP are detrimental to cell cycle progression. Networks with cancer-related functions had the highest significance, however reproductive networks containing follistatin and FSH were also significantly affected, which confirmed NASP's important role in reproductive tissues. This study revealed that, despite some overlap, each response was associated with a unique gene signature and placed NASP in important cell regulatory networks.

## Background

Nuclear autoantigenic sperm protein (NASP) is highly expressed in all dividing cells including embryonic and malignant tissues as either tNASP (testicular/embryonic isoform) or sNASP (somatic/embryonic isoform). Human tNASP contains three functional histone binding sites; sNASP is the shorter version of tNASP with two deletions in the coding region arising from alternative splicing, resulting in two histone binding sites. In embryonic and transformed cell lines both sNASP and tNASP are expressed and tNASP is present in a variety of malignant tumors [1]. Different cancer types as well as different stages of the same cancer demonstrate specific NASP RNA expression profiles, for example expression levels are up regulated in grade 1 and 2 versus grade 3 in breast cancer [2], estrogen receptor positive versus negative tumor types, or sporadic versus BRCA1/BRCA2 mutation positive tumors [3]. Thus, the NASP expression profile could be used to establish the "poor prognosis signature" which consists of genes regulating cell cycle, invasion, metastasis and angiogenesis [4]. Indeed, NASP has been reported as a serologic marker for ovarian cancer, which could be suitable for clinical testing in high-risk populations [5].

NASP has interactions with a variety of chromatin remodeling proteins: 1) linker H1 histones [1], 2) CAF1(p150, p60, p48) and HIRA in DNA synthesis dependent or independent nucleosome assembly pathways promoted by histone H3.1 and H3.3 complexes [6,7], 3) proteins involved in DNA repair (NASP is phosphorylated after DNA damage by irradiation of U2OS cells [8] and binds to Ku70/Ku80 and DNA PK in HeLa cells [9]). As a linker histone H1 chaperone, NASP binds linker histones in the cytoplasm and transports them into the nucleus [10], where NASP facilitates the incorporation of linker histones onto nucleosome arrays [11]. The present list of NASP interacting proteins is far from complete because Sun et al. [12] reported 356 network connectivity episodes for NASP in developing embryonic stem cells, suggesting numerous direct protein-protein interactions.

NASP is a tightly regulated cell cycle protein because both increased levels of NASP by overexpression [13] and decreased levels induced by siRNA treatment [14] cause disruption of the cell cycle. NASP mRNA levels increase during S-phase and decline during G_2_concomitant with histone mRNA levels [1] and NASP is required for cell survival because the NASP^-/- ^null mutation causes embryonic lethality [14]. Because HeLa cells have well studied signaling pathways and are easy to transfect, we chose them to study the general effect of increased or decreased NASP levels. Those networks and pathways that are involved in reproductive physiology were identified and will be further studied in reproductive tissues.

## Methods

### Materials

All chemicals and reagents used in this study were molecular biology grade. Restriction enzymes were purchased from Roche Applied Science (Indianapolis, Indiana, USA). Purification of plasmid DNA and PCR products was carried out using QIAprep Miniprep and QIAquick PCR purification kits (Qiagen, Valencia, California, USA); sequencing was performed at the University of North Carolina at Chapel Hill automated sequencing facility. Goat antiserum to full-length human recombinant tNASP (GenBank: AAH10105) was made by Bethyl Laboratories (Montgomery, Texas, USA).

### NASP overexpression

The entire coding sequence of mouse tNASP (nucleotides 92–2405, GenBank: AF034610) was amplified from mouse testis Quick-clone cDNA (Clontech, Palo Alto, California, USA) using the Expand High Fidelity PCR System (Roche Applied Science, Indianapolis, Indiana, USA) and cloned into a Kpn1/BamH1 site in the pEGFP-N1 vector (Clontech, Palo Alto, California, USA) which contains the sequence for the green fluorescent protein. To prevent possible effects of the expressed GFP [15], the GFP sequence was removed. All constructs were sequenced to verify the correct reading frame. Plasmid-DNA complexes were transiently transfected into HeLa cells using Effectene transfection reagent (Qiagen, Valencia, California, USA) according to the manufacturer's recommendations. This method is based on a non-liposomal lipid formulation, and resulted in low cytotoxicity and high transfection efficiency (~97%) as determined by FACS analysis [13]. Control cells were transfected with the transfection reagent only (Effectene transfection reagent, Qiagen, Valencia, California, USA). This choice of control cell treatment was based on the data that "mock" transfection with lipid formulation transfection reagents could consistently affect gene expression [16]. Transfection efficiency was confirmed by Western blotting: lysates from HeLa cells overexpressing tNASP were separated by SDS-PAGE, blotted and probed with goat anti-NASP affinity purified antibody.

### siRNA transfection

A series of siRNAs targeting the human *NASP *open reading frame were designed (Dharmacon, Lafayette, CO) and one (GCACAGUUCAGCAAAUCUAdTdT) was found to effectively deplete both tNASP and sNASP from HeLa cells [14]. Transfection with C2 siRNA, which had no cellular target, served as a negative control [17]. HeLa cells (8.5–10 × 10^5 ^cells per well in a 24-well plate) were transfected with NASP and C2 siRNA utilizing a two-hit siRNA transfection method with Lipofectamine™2000 for 18 hr as described [17]. Twenty four hours after the first transfection cells were trypsinized and split into 6-well plates. Forty eight hours after the first transfection cells were re-transfected. Ninety hours after the initial transfection cells were harvested for RNA purification.

### RNA isolation and hybridization of RNA to oligonucleotide arrays

Total cellular RNA was purified from HeLa cells using RNeasy^® ^Mini Kit (Qiagen, Valencia, California, USA) according to the manufacturers' instructions. RNA samples representing four separate experiments from cells overexpressing tNASP and four experiments from cells treated with NASP siRNA, along with appropriate controls, were submitted for analysis. After the RNA Quality check was performed the double-stranded cDNA was synthesized from RNA via MMLV reverse transcriptase. Amplified labeled cRNA was created via T7 RNA polymerase, which simultaneously amplifies the target material and incorporates Cy3- or Cy5-labeled CTP with at least a 100-fold RNA amplification rate. cRNA from treated cells was amplified with incorporation of Cy5-CTP (fluorescent in the red region), while cRNA from control samples was labeled by Cy3-CTP (fluorescent in the green region) and purified. cDNA synthesis, cRNA synthesis, amplification and labeling were done using the Low RNA Input Linear Amplification Kit (Agilent Technologies, Wilmington, Delaware, USA). The labeled cRNA samples were then fragmented in fragmentation buffer at 60°C for 30 min before the microarray hybridization. Each sample was hybridized to a whole separate Human Genome (4 × 44K) microarray (Agilent Technologies, Wilmington, Delaware, USA) overnight at 65°C in a hybridization oven. The hybridization slides were washed, stabilized, dried, and immediately scanned by Agilent Technologies Microarray Scanner (Agilent Technologies, Wilmington, Delaware, USA). RNA hybridization was performed in the Genomics and Bioinformatics Core Facility (Lineberger Comprehensive Cancer Center, UNC-CH) according to the protocol suggested by Agilent (Agilent Technologies, Wilmington, Delaware, USA).

### Statistical analysis

During the initial analysis at UNC Microarray Database (Genomics and Bioinformatics Core Facility, Lineberger Comprehensive Cancer Center, UNC-CH), all genes were retrieved, appropriately annotated, and filtered. Eventually, only genes with an absolute value of a Log_2 _Red/Green Lowess Normalized Ratio of at least 1 (doubled in intensity) for all 4 arrays were selected. The complete processed and raw data were deposited in Gene Expression Omnibus (GEO) and can be found as GSE14972 [18].

The UNC Microarray Database analysis generated a list of genes with an altered expression (at least 2 fold increased/decreased) between overexpression/depletion and mock treated samples. To identify which of these genes were significantly differentially expressed (significant genes) we used a statistical technique called SAM (Significance Analysis of Microarrays; [19]). SAM assigns a score to each gene on the basis of a change in gene expression relative to the standard deviation of repeated measurements. For genes with scores greater than an adjustable threshold, SAM uses permutations of the repeated measurements to estimate the percentage of genes identified by chance – the false discovery rate (FDR). Analysis parameters (Delta) were set to result in zero FDR.

To provide a rapid biological interpretation (from "genes to themes") of the obtained data, significant genes from SAM analysis were analyzed by the Expression Analysis Systematic Explorer (EASE). EASE calculates over representation with respect to the total number of genes assayed and annotated within each system to allow comparisons of categories from categorization systems [20]. We used three main categories: biological processes, cellular components, and molecular function. For each classification within the category the Fisher exact probability of over representation was calculated. Presented in this study the EASE score serves as a p-value to the Fisher exact probability that weights significance in favor of themes supported by more genes [20].

Functional interpretation of significant genes in the context of gene ontology, molecular networks and relevance to canonical pathways was generated through the use of Ingenuity Pathways Analysis (IPA 6.5 software, Ingenuity Systems^® ^[21]). Gene ontology analysis was based on an approach similar to EASE analysis, but the main categories used were in connection to top biological functions: diseases and disorders, molecular and cellular functions, and physiological system development and function. The significant genes were categorized, compared to genetic categories in the IPA database, and ranked according to p-values. P-values less than 0.05 indicate a statistically significant, non-random association between a set of significant genes and a set of all genes related to a given function in Ingenuity's knowledge base [22]. The IPA analysis determined the subcategories within each category supplied with an appropriate p-value and the number of genes identified.

The set of significant genes was used to find possible connections between genes/gene products and other genes based on interactions previously reported in the literature. Intermolecular connections were presented as molecular networks. Interacting genes (not found in SAM analysis but present in the IPA knowledge base) were used by IPA software to connect smaller groups of significant genes into a larger network. Since the size of the created network could potentially be enormous, the IPA software limited the number of molecules in the network to 35, leaving only the most important ones based on the number of connections for each focus gene (focus genes = a subset of uploaded significant genes having direct interactions with other genes in the database) to other significant genes [23]. Focus molecules and interacting molecules are presented separately. Networks are scored based on the number of focus molecules in the network, its size, the total number of focus molecules analyzed, and the total number of molecules in the knowledge database that could potentially be included in the networks [23]. The network score is the negative log of Fisher's Exact Test p-value. Only networks with a score of at least 10 (p-value of 10^-10^) were analyzed.

The final IPA analysis compared the list of significant genes with established pathways associated with metabolism and signaling (canonical pathway analysis). The results are presented in a diagram based on scoring and the ratio of significant genes present in the canonical pathway to the total number of molecules in the canonical pathway. The threshold level was set at p = 0.05. These analyses determined which pathways were involved under our experimental conditions.

## Results and discussion

### SAM analysis

**Figure 1:**
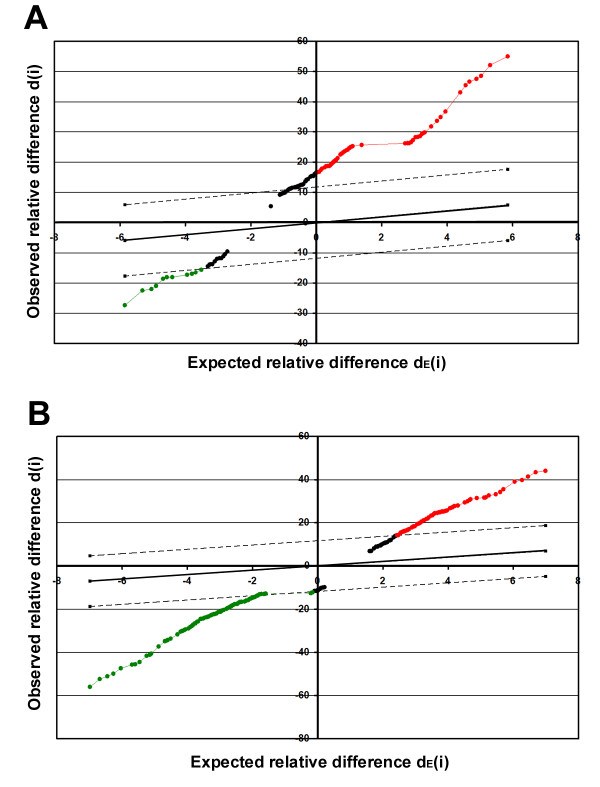
**SAM identification of genes with significant changes in expression**. **A**. Scatter plot of the observed relative difference *d*(*i*) versus the expected relative difference *dE*(*i*) in cells overexpressing NASP. The solid line indicates the line for *d*(*i*) = *dE*(*i*), where the observed relative difference is identical to the expected relative difference. The dotted lines are drawn at a distance Delta 11.79 from the solid line. **B**. Scatter plot of the observed relative difference *d*(*i*) versus the expected relative difference *dE*(*i*) in cells treated with NASP siRNA. The solid line indicates the line for *d*(*i*) = *dE*(*i*), where the observed relative difference is identical to the expected relative difference. The dotted lines are drawn at a distance Delta 11.75 from the solid line.

SAM analysis resulted in identification of groups of genes that were significantly differentially expressed after overexpression or depletion of NASP in HeLa cells at least two fold in all four experiments. Overexpression of tNASP in HeLa cells significantly affects the expression level of 54 genes (0.14% of 39,064 genes included in the microarray): 47 (0.12%) were up-regulated and 7 (0.02%) were down-regulated. Figure 1A shows a scatter plot summary of up-regulated and down-regulated genes from cells overexpressing NASP. Inhibition of NASP expression by siRNA treatment significantly affected the expression of 127 genes (0.32%): 56 (0.14%) were up-regulated and 71(0.18%) were down-regulated. Figure 1B shows a scatter plot summary of up-regulated and down-regulated genes from cells treated with NASP siRNA. The different ratios between up-regulated and down-regulated genes demonstrate that there was no bias between overexpression, siRNA treatment, and control groups due to dye affinity misbalance. The list of significant genes is presented in table 1.

**Table 1:** List of genes affected by NASP's altered expression

OVEREXPRESSION		siRNA TREATMENT	
Up-regulated	Down-regulated	Up-regulated	Down regulated
Function/Gene	Function/Gene	Function/Gene	Function/Gene
Transcription regulators: ATF3, DDIT3, ETV5, FOSL1, IRF7, IRF9, RELB, STAT1	Transcription regulators: EGR1, FOS, LMO1, NFE2	Transcription regulators: HMGB2, E2F8	Transcription/translational regulators: SRF, HOXA10, EIF2C4
	Transporters: AQP5	Enzymes: ARL5A, CYP1B1, ME3, GSR, MANEA, GNG2, OGG1	
Enzymes: BIRC3, DDX60, IFIH1, OAS1, OAS3, OASL, PTGS2, RRAD	Others: KR19, PLCXD3	Ion channel: KCNJ15	Enzymes: ACADL, ACADSB, GLS, ARL1, CROT, DHTKD1, ENTPD7, FAAH2, GBP3, GATM, LIPH, SEPX1, ZDHHC2, UBE2N, SCCPDH
Cytokines: CXCL1, CXCL2, CXCL3, TNF, IL11, IL1A, IL6, IL8		Kinases: PI4K2B, PDGFRB	Kinases: MAP2K1, RFK
Growth factors: DKK1, EREG, GDF15		Transmembrane receptors: TNFRSF11B	Transmembrane receptors: ITGB3, TLR3, ITGA5
			Growth factors: FGF2
Phosphatases: DUSP5, NT5E		G-protein coupled receptors: F2RL1	Peptidases: CFI, CPA4
G-protein coupled receptors: MRGPRD		Transporters: ATP6V1G3, FMN1, PNMA2, SMC1A, SAA1, SLC25A32	Transporters: ATP11A, ATP2B4, FABP5, NUPL1, SEC13, SLC25A43
			Phosphatases: PPM1K
Others: CLDN1, TNFAIP3, ZC3H12A, HRK, IFI27, IFI6, IFITM1, IGFL2, KRT34, LOC283454, PARP12, PARP9, PHLDA1, PPP1R15A, SAMD9L, STC2, GADD45A		Others: ARL6IP6, C14ORF167, CALB1, CALML4, CGA, CCDC5, CRYAB, FSTL1, FGFBP3, PRR16, SSU72, DTWD2, FST, ZMYM6, HAPLN1, TMSL8, MKX, HCG1815491, KIF5C, SYTL5, MFAP5, PSIP1, LOC100130476, RDM1, MEX3C, PHACTR2, NAP1L5, TMEM64, RASSF8, SLITRK6, ZBED2, MEFF2, TES, RBM17, TMEM80, UBE2E3	Others: CPEB3, DCBLD2, DCP2, DENND1B, TMEM9B, TNC, ERLIN2, EVI5, GCOM1, HIST1H1C, HIST1H2BK, ZFYVE26, HIST2H2AA3, ZBTB41, NASP, CDCP1, KIAA0329, LOC285535, LONRF1, GPR137B, OSTM1, PCDH7, PPP1R3B, TM9SF4, PPP1R9A, PRRG4, SLAIN2, TUBB3, SHOC2, GLT8D3, CNN1, SPOCD1, DYX1C1, TTC39A, ACTA1, ACTA2, CCDC109A, CCDC126

### EASE analysis

All genes reported in table 1 were subjected to Gene Ontology (GO) clustering by EASE software [20]. EASE analysis is presented in table 2 and table 3. Themes with a p value of < 0.05 and with at least three genes in the category are reported [24].

**Table 2:** Gene ontology categories significantly (p < 0.05) up-regulated and down-regulated in HeLa cells overexpressing NASP

UP REGULATED		DOWN REGULATED	
P-value	GO category	P-value	GO category
1.56e-011	immune response	6.75e-003	transcription regulator activity
2.24e-011	response to biotic stimulus	2.68e-002	transcription factor complex
7.52e-011	defense response	3.85e-002	transcription factor activity
5.30e-009	response to external stimulus	4.21e-002	DNA binding
1.16e-008	cytokine activity	4.75e-002	nucleoplasm
5.89e-008	receptor binding		
7.08e-007	response to stress		
1.12e-006	response to pest/pathogen/parasite		
1.62e-006	inflammatory response		
1.81e-006	response to wounding		
2.39e-006	innate immune response		
8.28e-006	growth factor activity		
9.48e-005	response to chemical substance		
1.96e-004	regulation of cell proliferation		
3.26e-004	apoptosis		
3.31e-004	regulation of cell cycle		
3.36e-004	programmed cell death		
3.46e-004	chemokine activity		
3.46e-004	chemokine receptor binding		
4.59e-004	cell death		
4.87e-004	death		
5.27e-004	cell proliferation		
7.09e-004	regulation of cellular process		
7.72e-004	regulation of biological process		
8.20e-004	cell cycle arrest		
9.52e-004	regulation of cell proliferation		
1.83e-003	cell-cell signaling		
3.38e-003	response to virus		
3.38e-003	anti-apoptosis		
7.00e-003	cell communication		
7.10e-003	signal transducer activity		
9.37e-003	cell cycle		
1.15e-002	response to abiotic stimulus		
1.61e-002	nucleic acid binding		
2.58e-002	transcription factor activity		
3.10e-002	transcription regulator activity		
3.27e-002	response to DNA damage stimulus		
3.27e-002	regulation of apoptosis		
3.31e-002	response to endogenous stimulus		

**Table 3:** Gene ontology categories significantly (p < 0.05) up-regulated and down-regulated in HeLa cells treated with NASP siRNA

UP REGULATED		DOWN REGULATED	
P-value	GO category	P-value	GO category
1.10e-003	morphogenesis	1.51e-002	DNA packaging
2.10e-003	organogenesis	1.59e-002	cell adhesion receptor activity
7.68e-003	chromosome	1.67e-002	fatty acid metabolism
1.00e-002	nucleic acid binding	1.73e-002	muscle development
1.22e-002	chromatin	2.54e-002	carboxylic acid metabolism
1.36e-002	nucleic acid metabolism	2.95e-002	nucleosome assembly
1.69e-002	development	3.04e-002	chromatin architecture
2.02e-002	response to radiation	3.18e-002	nucleosome
2.11e-002	DNA packaging	3.33e-002	acyl-CoA dehydrogenase activity
3.19e-002	extracellular space	3.63e-002	chromosome organization
4.64e-002	intracellular transporter activity	3.90e-002	nuclear organization and biogenesis
4.84e-002	DNA binding	4.25e-002	DNA metabolism
4.96e-002	transporter activity	4.39e-002	chromatin assembly/disassembly
		4.73e-002	chromatin
		4.79e-002	development
		4.87e-002	chromosome

In HeLa cells overexpressing tNASP, EASE identified 39 up-regulated and 5 down-regulated GO categories (table 2). Fourteen percent of up-regulated genes are located on chromosome 4. In the group of up-regulated genes the highest EASE score as well as the highest number of genes was found within the categories represented by proteins participating in the immune response, the response to biotic and external stimuli, stress, and to pathogens. These results may indicate a rather non-specific reaction of HeLa cells to an excessive amount of expressed recombinant NASP. Down-regulated genes in NASP overexpressing cells represented proteins involved in transcription regulator activity, transcription factor complex structure and activity, and nucleoplasm structure.

Gene ontology categories affected in cells treated by siRNA are presented in table 3. Most of the up-regulated genes in this category are located on chromosome 1(17%), the same chromosome where the NASP gene is located. In cells with inhibited NASP expression, up-regulated categories (n = 13) represented gene products that were involved in morphogenesis and organogenesis, chromosome organization (chromosome, nucleic acid binding, chromatin, DNA packaging, DNA binding), development, and transporter activity. Down-regulated genes in NASP deficient cells represented a group of proteins involved in DNA packaging, nucleosome structure and assembly, establishment and maintenance of chromatin architecture, DNA metabolism, chromosome organization and biogenesis, chromatin assembly/disassembly, and chromatin/chromosome structure (a total of 16 categories). This result is consistent with earlier observations that histone H1 transfer between NASP and DNA affects chromatin structure [13].

Although EASE analysis identified general categories, it did not provide detailed subcategories or connect identified genes to discrete disorders and functions. Therefore additional functional GO analysis was carried out employing the IAP 6.5 software.

### IPA analysis

#### Top bio functions

**Figure 2:**
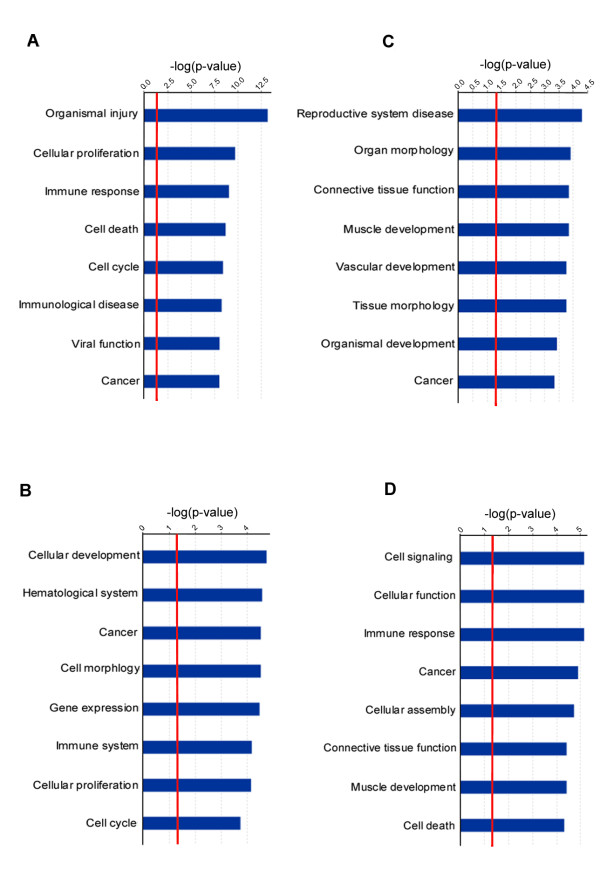
**Top functions affected as a result of altered gene expression**. **Functions determined by**: **A**. Up-regulated genes after NASP overexpression. **B**. Down-regulated genes after NASP overexpression. **C**. Up-regulated genes after NASP depletion. **D**. Down-regulated genes after NASP depletion. Bars represent -log (p-value) for disproportionate representation of affected genes in the total number of genes in the selected function/disease category. Threshold (red line) denotes the p = 0.05 level.

Genes with altered expression in both NASP overexpression and inhibition experiments were analyzed by IPA software. The analysis of the top eight affected functional categories (highest significance) is presented in figure 2. Each group of significant genes (A: up-regulated after overexpression; B: down-regulated after overexpression; C: up-regulated after siRNA treatment; D: down-regulated after siRNA treatment) was over represented in the set of genes related to some function or disease category. The top affected subcategories are presented in Additional file 1, Additional file 2, Additional file 3 and Additional file 4.

##### NASP overexpression

The highest scoring category in NASP overexpressing cells was "organismal injury", subcategory "fibrosis" (p-value 6.35 × 10^-14^, represented by 13 genes: ATF3, IFI6, IFI27, IFITM1, IL6, IL11, IRF7, IRF9, NT5E, OAS1, PTGS2, STAT1, TNF). A high number of up-regulated genes were functionally related to the "cell growth" subcategory, part of the category "cellular proliferation" (22 molecules out of 47 analyzed, including transcription factors ATF3, DDIT3, FOSL1, RELB, STAT1, cytokines CXCL1, CXCL2, CXCL3, TNF, IL6, IL8, IL11, IL1A, and growth factors EREG, GDF15). The "apoptosis of eukaryotic cell" subcategory of the "cell death" category was represented by an overlapping with the "cell growth" set of genes (total= 24 genes). The most over represented subcategories in the "cell cycle category" were "cell division process of cells "and "arrest in cell division process of cells" with 18 and 12 molecules respectively. The category "cancer" had the highest number of genes (30 out of 47 analyzed). The leading number of molecules (16) was in the subcategory "developmental process of tumor cell lines" and "apoptosis of tumor cell lines". "Immune response", "immunological disease", and "viral functions" categories were selected as a result of the increased expression of a group of cytokines: CXCL1, CXCL3, IL6, IL8, IL11, and TNF.

Down-regulated genes in NASP overexpressing cells were over represented in a group of interrelated functions (figure 2B) mostly as a result of down-regulation of 4 significant genes: transcription regulators EGR1, FOS, LMO1, and NFE2. Their changed expression affected subcategories related to differentiation, maturation, and development within such categories as cellular development (subcategory "developmental process of blood cells", p-value 1.25 × 10^-4^), hematological system (subcategory "differentiation of blood cells", p-value 2.67 × 10^-5^), cell morphology (subcategory "morphology of tumor cells", p-value 3 × 10^-5^), immune system (subcategory "development of macrophages", p-value 6.69 × 10^-5^), gene expression (subcategory "transactivation", p-value 2.1 × 10^-3^). All these categories are interrelated and the different level of significance is a reflection of the total number of genes in each category. The subcategory "arrest in G_0_/G_1 _phase of eukaryotic cells" from category "cancer" had the highest significance (p-value of 1.82 × 10^-4^) within a given category.

##### NASP inhibition

Although up-regulated genes after siRNA treatment identified a list of discrete and diverse functional categories (figure 2C), the number of molecules presented in each subcategory was low (1–4). Therefore these categories and subcategories were assigned a low significance. In the "reproductive system disease" category the presence of transcription regulator HMGB2 determined the subcategories connected to the degeneration of Sertoli cells (p-value 2.93 × 10^-3^), testicular cells (p-value 2.93 × 10^-3^), and germ cells (p-value 2.03 × 10^-2^). Follistatin (FST) and CGA (alpha subunit of glycoprotein hormones) identified the categories related to degeneration of seminiferous tubules (p-value 4.98 × 10^-5^), disease process of testicular cells (p-value 1.38 × 10^-3^), infertility (p-value 6.67 × 10^-3^), and several organ morphology subcategories (size of the organ, CALB1, CGA, FST, KIF5C, p-value 7.98 × 10^-4^). Transmembrane receptor TNFRSF11B determined the selection of multiple subcategories associated with bone mineral density (p-value 1.42 × 10^-4^) within the "connective tissue function" category as well as myogenesis (p-value 2.89 × 10^-2^) within the "muscle development" category. Subcategories related to vascular development were determined by combined up-regulation of G-protein coupled receptor F2RL1 and growth factor receptor PDGFRB. In the cancer category cytochrome P450 (CYP1B1) and PDGFRB identified the endometrial cancer (p-value 1.37 × 10^-2^) and endometrial carcinoma (p-value 1.68 × 10^-2^) subcategories. Testis derived transcript (TES) was present in subcategories tumorigenesis, breast cancer, and neoplasia. All 5 genes (CYP1B1, FST, HMGB2, PDGFRB, and TNFRSF11B) were present in the "colorectal cancer" subcategory.

After siRNA treatment down-regulated transmembrane receptors ITGB3, ITGA5, and TLR3 determined the functional categories "cell signaling", "cellular function", "immune response", and "cancer" with subcategories related to phagocytosis, adhesion, and binding. Down-regulated transcription factor SRF (serum response factor) and FGF2 (fibroblast growth factor 2) are reported in categories "cellular assembly", "cell death" and "muscle development". Seventeen molecules are identified in the subcategory "tumorigenesis" and "neoplasia" within the "cancer" category. Along with transcription factor HOXA10 and transmembrane receptors TLR3 and ITGB3, other molecules ACTA2, CFI, CNN1, CPA4, DHTKD1, FABP5, FGF2, HIST2H2AA3, ITGA5, LIPH, MAP2K1, TNC, TUBB3, ZFYVE26 were present in these subcategories.

#### Network analysis

##### NASP overexpression

Network analysis assembled three networks from up-regulated genes after NASP overexpression (table 4A). The network with the highest score (table 4A, network #1) included gene products associated with TNF, which is a multifunctional proinflammatory cytokine that belongs to the tumor necrosis factor (TNF) superfamily. This cytokine is involved in the regulation of a wide spectrum of biological processes including cell proliferation, and differentiation, and has been implicated in cancer and autoimmune diseases [25]. In response to TNF and growth factors, STAT1 protein (found up-regulated in NASP overexpressing cells), is phosphorylated and translocates to the nucleus where it acts as a transcription activator [26], mediating the expression of a variety of genes. In this network ATF3 (activating transcription factor) is connected with TNF, which is known to increase the expression of human ATF3 mRNA in LoVo cells [27].

**Table 4:** The highest scoring networks after NASP overexpression

	A. UPREGULATED GENES			
	Focus genes	Interacting genes	Score	Top functions

1	ATF3, CXCL2, DUSP5, GDF15, IFI6, IFI27, IFIH1, IFITM1, IRF7, IRF9, KRT34, OAS1, OAS3 (includes EG:4940), OASL, PARP9, RELB, STAT1, TNF, TNFAIP3	Cyclooxygenase, IFN Beta, Ifn gamma, IL1/IL6/TNF, Interferon alpha, Interferon beta, IRF, ISGF3, LDL, MHC Class I, NF-κB, NfkB-RelA, SAA@, Sod, Stat1-Stat2, Tlr	44	organismal injury and abnormalities, gene expression, immune response
				
2	CXCL1, CXCL3, DDIT3, DKK1, EREG, OSL1, IL8, L11, IL1A, PHLDA1, PTGS2, PPP1R15A (includes EG:23645)	Akt, ALP, Ap1, Cbp/p300, Creb, ERK, hCG, Hsp27, Hsp90, IKK, IL1, JAK, MAP2K1/2, Nos, P38 MAPK, Pdgf, PDGF BB, PI3K, Pkc(s), PLC, STAT, Tgf beta, Vegf	25	cellular growth and proliferation, cellular movement, hematological system function
				
3	BIRC3, ETV5, GADD45A, IL6, RRAD, STC2	ABLIM, ADCY, CAP2, Ck2, Caspase, Cytochrome C, FSH, DYRK3, Histone h3, Hsp70, IL12, IL1/IL6/TNF, IL1F8, IL1F9, Insulin, Jnk, Mapk, Nfat, NFkB, Pka, Proteasome, Rac, Ras, RNA polymerase II, STAT5a/b, UBR2, Vacuolar H+ ATPase, ZNF274, ZNF675	10	infectious disease, cell cycle, cancer

	B. DOWNREGULATED GENES			

	Focus genes	Interacting genes	Score	Top functions

1	AQP5, EGR1, FOS, KRT19, LMO1, NFE2, TNF	ADCYAP1, AKR1B10, ARF4, ASC2, BARX2, BPI, CCL9, COBRA1, CXCL16, DGCR6, EMB, FOXF1, GFPT2, HMBS, IL1/IL6/TNF, IL1F9, JUN, LOC729687, LTBP2, MFHAS1, NFkB, RFTN1, SFI1, SLC7A1, TNIP3, TRAFD1, Vacuolar H+ ATPase, WNT10A	17	gene expression, cell cycle, cellular development

The other two networks (table 4A, network #2 and #3) contained groups of interacting proteins that included GADD45A, interleukins IL6, IL1A, chemokines CXCL3, CXCL1, and NF-κB. NF-κB is a transcription factor that mediates the transcription of proteins involved in cell survival, proliferation, and inflammatory responses, and is the subject of active research for anti-cancer therapy [28]. The protein encoded by the GADD45A gene induces apoptosis and cell cycle arrest by maintaining p38 and c-JNK MAPK activation in keratinocytes. The absence of Gadd45a results in loss of sustained p38/JNK MAPK activity that leads to inadequate p53 activation and loss of normal activation of G_1 _and G_2 _checkpoints [29].

Only one network was assembled from the down-regulated genes after NASP overexpression (table 4B). It included TNF, FOS, LMO1, EGR1, NFE2, KRT19, and AQP5. TNF, FOS, and NFκB are the "hubs" that suggests possible involvement of this network in cell proliferation, differentiation, and transformation. The presence of transcriptional regulator EGR suggests cancer suppressor activity [30].

##### NASP inhibition

Distinct networks were assembled with up-regulated genes after NASP siRNA treatment (table 5). The highest scoring network (table 5, network #1) presents E2F8 interacting with TP53. E2F8, which is in the family of E2F transcription factors, is essential for orchestrating expression of genes required for cell cycle progression and proliferation [31]. In the assembled network E2F8 interacts with tumor protein p53 [32], which wasn't found as a focus gene in this study, but was added during network assemblage as an interacting gene. Protein p53 regulates target genes that induce cell cycle arrest, apoptosis, senescence, DNA repair and is postulated to function as a tumor suppressor [33]. Multiple focus genes TMSL8, OGG1, HMGB2, GSP, ARL5A, which were reported to be acted upon by p53 [34-39], were found to be up-regulated in this study. One of them, NAP1 (nucleosome assembly protein1) plays a role in chromatin maintenance by facilitating core histone exchange (by regulating the concentration of free histones) as well as nucleosome assembly and disassembly [40].

**Table 5:** The highest scoring networks after NASP siRNA treatment (upregulated genes)

	Focus genes	Interacting genes	Score	Top functions
1	ARL5A, E2F8, GSR, HMGB2, NAP1L5, OGG1, PSIP1, RBM17, RDM1, TMEFF2, TMSL8, UBE2S	APOBEC1, ERBB4, Erbb4 dimer, ERBB4 ligand, ESR2, HCFC1, HINT1 (includes EG:3094), IGH@, KAT5, KPNA2, MAD2L1BP, MAGEH1, NRG3, NRG4, POLE2, RAG1, RCHY1, TBXAS1, TP53, UBE2E3, UBE2V1, WRN (includes EG:7486), WWOX	25	DNA replication, recombination, and repair, cellular development, connective tissue disorders
				
2	CALB1, CGA, F2RL1, FST, FSTL1, GNG2, SAA1, SYTL5, TNFRSF11B, ZBED2	AKR1C14, ARHGAP22, ARHGEF5, ATP9A, C5ORF23, CENPI, ERK, FJX1, FSH, GK7P, hCG, IL2, LOC81691, LOC652955, MARCH3, NFkB, MRPS6, PI4K2A, RAB27A, REGL, STEAP1, Tgf beta, TP53I11, ZFP386, ZNF808	20	reproductive system disease, respiratory disease, immune response
				
3	TES, CRYAB, HAPLN1, KIF5C, MFAP5, PDGFRB, PI4K2B, PNMA2, RASSF8, CCDC5	AASS, Abl1/2, Cadherin (E, N, P, VE), CAPRIN1, CDKN2A, CNN2, CTNNB1, DLL3, FRMD6, GLRX2, hydrogen peroxide, KIFC1, KLC3, KRT1, LSR, MLXIP, NOTCH1, PMEPA1, PXN, SRC, SRFBP1, TAX1BP3, TGFB1, TOB2, YWHAG	20	cancer, cell death, cell cycle

Network #2 (table 5) of genes up-regulated after siRNA treatment was assembled with the "hub" gene products FSH (follicle stimulating hormone), FST (follistatin), NFκB, hCG (human chorionic gonadotropin) and focus gene products TNFRSF11B, SYTL5, ZBED2, FSTL1, CALB1, GNG2, SAA1, F2RL1. Urbanek et al. [41] studied 37 candidate genes for linkage and association with polycystic ovary syndrome (PCOS) or hyperandrogenemia and found evidence for linkage between PCOS and follistatin. Follistatin has been reported as a modulator of gonadal tumor progression in inhibin deficient mice [42].

Network #3 (table 5) of up-regulated genes after siRNA treatment identified TGFB1 as a core molecule indirectly affiliated with interacting proteins. TGFB is a multifunctional peptide that controls proliferation, differentiation, inducing transformation, and other functions in many cell types [43].

After siRNA treatment 71 down-regulated genes were almost evenly assembled into four networks of interacting proteins (table 6). The network with the highest score (table 6, network #1) included: HOXA10, FGF2, SRF, ITGA5, and ITGB3. HOXA10 is a DNA-binding transcription factor that may regulate gene expression, morphogenesis, and differentiation. More specifically, it may function in fertility and embryo viability [44]. FGF2 (fibroblast growth factor) and SRF (c-fos serum response element-binding transcription factor) participate in cell cycle regulation, apoptosis, cell growth and differentiation [45]. Interaction of SRF with other proteins, such as steroid hormone receptors, may contribute to regulation of muscle growth [46].

**Table 6:** The highest scoring networks after NASP siRNA treatment (downregulated genes)

	Focus genes	Interacting genes	Score	Top functions
1	ACTA1, ACTA2 (includes EG:59), ARL1, ATP2B4, CNN1, DCBLD2, FGF2, HOXA10, ITGA5, ITGB3, MAP2K1, SEC13, SHOC2, SRF, TNC	Actin, Akt, Alpha actin, ERK, ERK1/2, FSH, IKK, Integrin, Jnk, Mapk, Mek, Pdgf, PDGF BB, PI3K, Pkc(s), PLC gamma, Pld, Ras, Tgf beta, Vegf	31	cellular assembly and organization, cell-to-cell signaling and interaction, viral infection
				
2	CDCP1, CROT, DYX1C1, ERLIN2, GCOM1, GLS, GPR137B, HIST2H2AA3, PPM1K, SLAIN2, SPOCD1, TTC39A, TUBB3, ZDHHC2	beta-estradiol, F2, HSPA1A, HSPA1L, IFRD2, KYNU, MAPK11 PREDICTED, VCP, MFHAS1, MMD, NFRKB, NOS3, PGLYRP1, SAMD4A, SLC16A5, SPR, TNF, TXN2, YWHAZ, ZDHHC8, ZNF267	28	cardiovascular system development and function, tissue morphology, organismal development
				
3	ACADL, ACADSB, CPEB3, ENTPD7, EVI5, CFI, CPA4, TLR3 GATM, LIPH GBP3, RFK, PPP1R9A, OSTM1	9330129D05RIK, ACAD8, ACAD9, ACAD10, ACAD11, Acyl-CoA dehydrogenase, C3, CDKN2A, CFHR3, CFHR5, F Actin, Glycogen synthase, GTP, heparin, IL4, IWS1, NFkB, NFYB, Pka, PLK1, RAB1A	28	lipid metabolism, molecular transport, small molecule biochemistry
				
4	DCP2, FABP5, GLT8D3, HIST1H1C, HIST1H2BK, NASP, NUPL1, PCDH7, PPP1R3B, SEPX1, TM9SF4, UBE2N	ANXA9, DHX8, ERLIN1, FASTKD2, HNF4A, IL1B, MGEA5, MRTO4, NAT13, NUDT11, NUP62, OTUD7B, PHPT1, PSMC3, REXO2, SEC11A, MEM176B, TMEM189-UBE2V1, TRAF6, TRAFD1, Ube2n-Ube2v1, UHRF1, WRNIP1	23	cell signaling, cancer, cellular compromise

**Figure 3:**
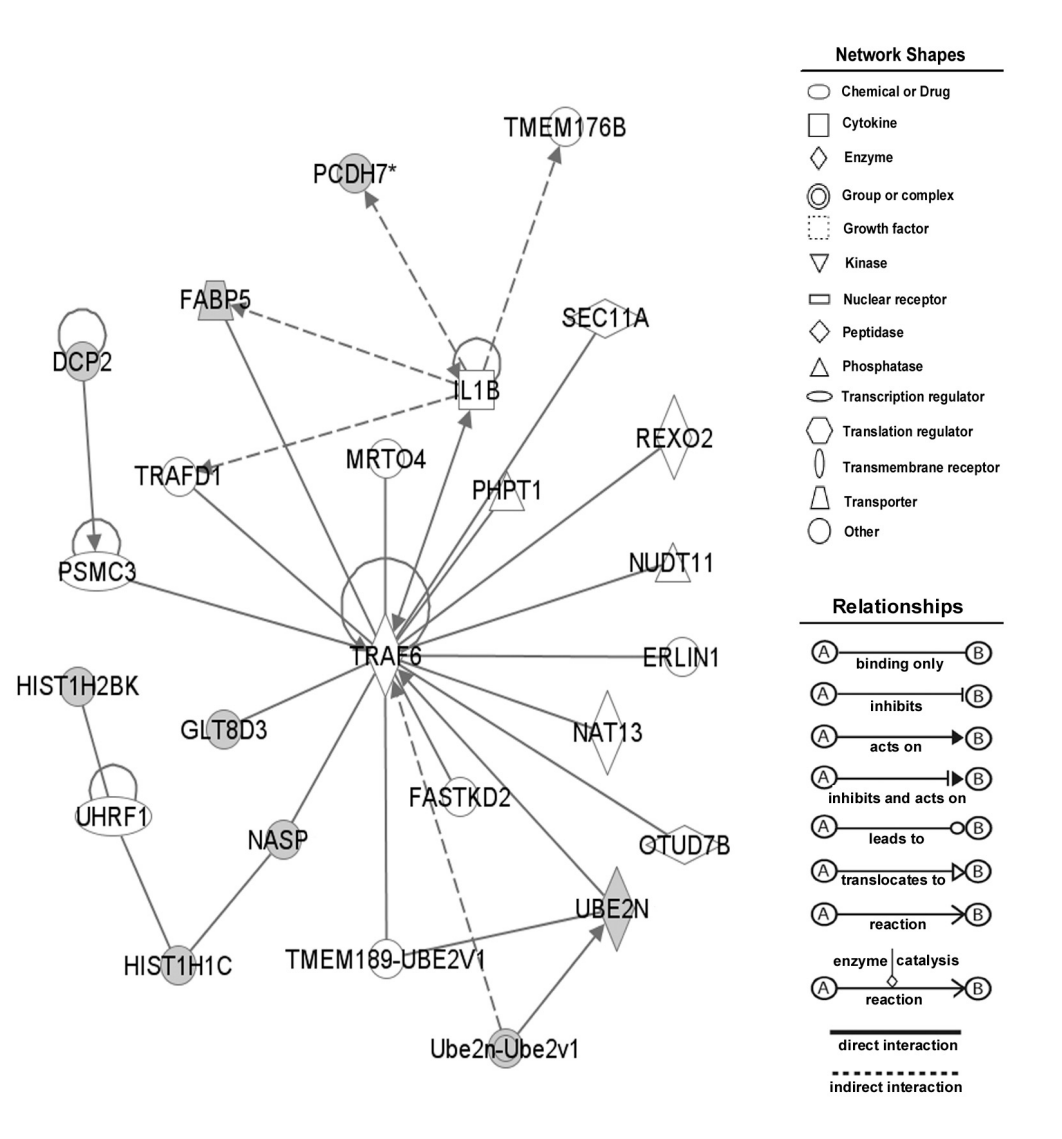
**Fragment of molecular network of down-regulated genes in the result of NASP depletion**. Shaded shapes present focus genes, clear shapes present interacting genes. Table 6 contains all the genes.

Network #2 of down regulated genes (table 6) includes TNF as a core protein along with several interacting proteins. Overlap of this network with another assembled network (table 6, network #4) revealed TRAF6 (TNF receptor associated factor), which mediates signal transduction from members of the TNF receptor superfamily and interacts with various protein kinases including IRAK1/IRAK, SRC and PKCzeta [47]. TRAF6 was identified by mass spectrometry in a large scale immunoprecipitation as an interacting protein with NASP protein, but the functional correlation between TRAF6 and NASP is unknown [48]. TRAF6 mediates signals from TNF apoptotic pathways, as well as other pathways (IL1, NF-κB). It is not clear if NASP regulates TRAF6, or TRAF6 transfers a signal to NASP. The focus genes interacting with NASP (table 6, network #4; figure 3) include HIST1H1C [1] and HIST1H2BK [49], which implies that these histone gene products may be regulated in concert with NASP. None of the focus genes were found to be a hub in network #3 (table 6), therefore it was not investigated further.

#### Canonical pathway analysis

##### NASP overexpression

We found that each treatment resulted in activation/inhibition of specific signaling pathways. A list of the significant genes present in canonical pathways after different treatments is presented in Additional file 5, Additional file 6, Additional file 7 and Additional file 8.

**Figure 4:**
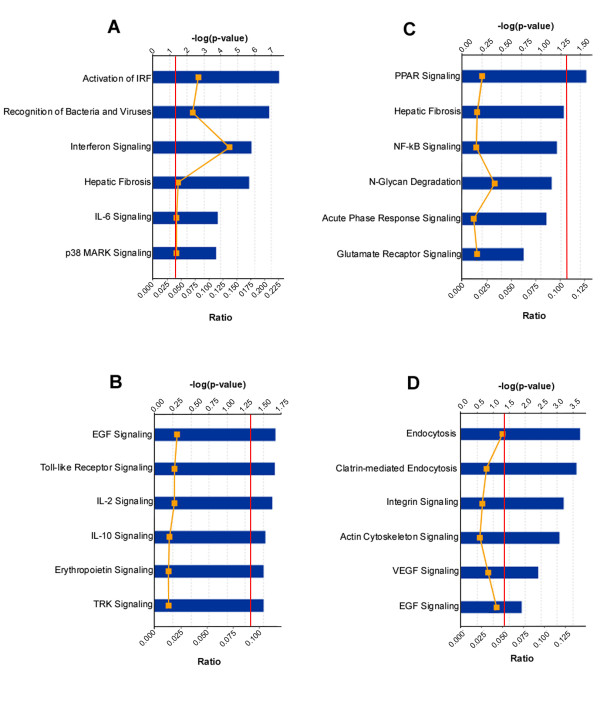
**Top canonical pathways affected by**. **A**. Up-regulated genes after NASP overexpression. **B**. Down-regulated genes after NASP overexpression. **C**. Up-regulated genes after NASP depletion. **D**. Down-regulated genes after NASP depletion. Bars represent -log (p-value) for disproportionate representation of affected genes in the selected pathway, yellow line represents the ratio of affected genes to the total number of genes in a pathway. Threshold (red line) denotes the p = 0.05 level.

The highest activation level after NASP overexpression (figure 4A) was detected in pathways related to antiviral responses, activation of IRF (interferon activation factor), recognition of bacteria and viruses, interferon signaling and other interrelated pathways. Some pathways were inhibited by NASP overexpression (figure 4B). The significance of all top 6 canonical pathways in this category barely exceeded the threshold level and all of them were selected as a result of down-regulation of only one gene, FOS, which is an important transcription regulator of cell proliferation, differentiation, and transformation [50]. The inhibition of a single gene makes understanding its role in any of those pathways difficult.

##### NASP inhibition

A similar result was observed with analysis of activated signaling pathways after siRNA treatment (figure 4C). Only one pathway reached significance above the threshold level, the remaining five canonical pathways were below the threshold level. After siRNA treatment (figure 4D) several signaling pathways were inhibited due to down-regulation of growth factor FGF2, transmembrane receptors ITGB3 and ITGA5 and other molecules such as ATCA1 and ATCA2. The two highest scores had canonical pathways related to caveolar-mediated (p-value 1.85 × 10^-4^, 4 out of 81 molecules were associated with a given pathway) and clathrin-mediated (p-value 2.39 × 10^-4^, 5/165) endocytosis. Selection of these pathways was based on down-regulation of both α- and β- integrins (ITGA5 and ITGB3) which serve as transmembrane receptors. Activation of α- and β-integrins by appropriate ECM proteins causes the activation of ERK/MAPK-mediated transcription, which eventually leads to cell proliferation [51]. Mitogen activated protein kinase MAP2K1 (synonym MEK1/2), an important link in both those pathways, was found to be down regulated and it makes NASP's effect on this pathway plausible. Down regulation of ACTA1 and ACTA2 (actin α-1 and actin α-2) along with MAP2K1, α-integrin, and FGF2 (fibroblast growth factor 2) indicates a high probability that there is an effect on the actin cytoskeleton signaling pathway (p-value 8.17 × 10^-4^, 5/222), which leads to actin reorganization and plays an important role in cell motility, cytokinesis and phagocytosis [52]. Decreased expression of kinase MAP2K1 and transcription factor SRF implies an effect on EGF (epidermal growth factor) signaling pathways (p-value 1.25 × 10^-2^, 2/47), which regulate cell growth and differentiation [53].

## Conclusion

This study has demonstrated that NASP, a linker histone chaperone, belongs to a network of genes and gene functions that are critical for cell survival. We have confirmed the previously reported interactions between NASP and HSP90, HSP70, histone H1, histone H3, and TRAF6. Indeed, based on the number of critical pathways affected by the overexpression or inhibition of NASP expression, NASP may play a much wider role in gene expression events that require the participation of histones. Significantly this study found that during overexpression the network with the highest score of up-regulated genes included gene products associated with TNF and during inhibition network #2 of down regulated genes also included TNF. TNF receptor associated factor, TRAF6, has already been identified as interacting with NASP. Therefore these two identified networks may explain how the expression of NASP is modulated during cell proliferation and differentiation.

Of interest to reproductive biology, this study found a significant association of NASP with the FSH and follistatin gene pathways that are up-regulated after NASP siRNA treatment. This may have implications for the control of NASP expression during granulosa cell and endometrial cell proliferation.

Most of the pathways found in this study have not been previously reported to be connected to expression levels of NASP and demonstrate a rather complicated picture of changed gene expression in HeLa cells after NASP expression was increased or decreased. Gene ontology and protein network analysis identified general biological processes as well as individual genes/gene products and possible interaction networks. Some of these processes may relate to HeLa-type cells in tissue culture in which cell adhesion and migration are critical, while others may only be relevant in neoplasia. We found signaling pathways which were affected as a result of changed NASP expression and despite some overlap each reactive response was associated with a unique gene signature. The results of this study have elucidated the changes that emerge from increased and decreased NASP expression and will help our understanding of the molecular mechanisms involved in NASP function. Confirmation of NASP's role in regulating the cell cycle may contribute to the development of new pharmaceutical approaches to control the relevant pathological conditions.

## Abbreviations

FACS: fluorescent-activated cell sorting; SDS-PAGE: sodium dodecyl sulfate polyacrylamide gel electrophoresis; cDNA: complementary DNA; cRNA: RNA derived from cDNA; siRNA: short interfering RNA; ECM: extracellular matrix.

## Competing interests

The authors declare that they have no competing interests.

## Authors' contributions

OMA conceived of the study, carried out the microarray statistical analysis and drafted the manuscript. RR assisted with experimental design and conceived of the study. OA performed the cell culture experiments: overexpression and siRNA treatment. MO'R conceived of the study, participated in project design and coordination, helped to draft the manuscript. All authors read and approved the final manuscript.

## Supplementary Material

Additional file 1 **Functions: overexpression, up-regulated genes**. The data provided represent the top affected subcategories determined by up-regulated genes after NASP overexpression. Click here for file

Additional file 2 **Functions: overexpression, down-regulated genes**. The data provided represent the top affected subcategories determined by down-regulated genes after NASP overexpression. Click here for file

Additional file 3 **Functions: siRNA treatment, up-regulated genes**. The data provided represent the top affected subcategories determined by up-regulated genes after NASP siRNA treatment. Click here for file

Additional file 4 **Functions: siRNA treatment, down-regulated genes**. The data provided represent the top affected subcategories determined by down-regulated genes after NASP siRNA treatment. Click here for file

Additional file 5 **Canonical pathways: overexpression, up-regulated genes**. The data provided represent the list of up-regulated genes present in top canonical pathways after NASP overexpression. Click here for file

Additional file 6 **Canonical pathways: overexpression, down-regulated genes**. The data provided represent the list of down-regulated genes present in top canonical pathways after NASP overexpression. Click here for file

Additional file 7 **Canonical pathways: siRNA treatment, up-regulated genes**. The data provided represent the list of up-regulated genes present in top canonical pathways after NASP siRNA treatment. Click here for file

Additional file 8 **Canonical pathways: siRNA treatment, down-regulated genes**. The data provided represent the list of down-regulated genes present in top canonical pathways after NASP siRNA treatment. Click here for file
